# Moscatilin Inhibits Metastatic Behavior of Human Hepatocellular Carcinoma Cells: A Crucial Role of uPA Suppression via Akt/NF-κB-Dependent Pathway

**DOI:** 10.3390/ijms22062930

**Published:** 2021-03-13

**Authors:** Chen-Lin Yu, Meng-Shih Weng, Wei-Cheng Chen, Kai-Ting Chien, Chih-Wen Chi, Ching-Hu Chung, Chia-Wen Huang, Po-Chuan Wang, Chien-Chih Chen, An-Chi Tsai, Shih-Chia Liu, Shih-Wei Wang

**Affiliations:** 1 Institute of Biomedical Sciences, MacKay Medical College, New Taipei City 252, Taiwan; p01753-506@mmc.edu.tw (C.-L.Y.); s74747412001@gmail.com (C.-W.H.); 2 Department of Medicine, MacKay Medical College, New Taipei City 252, Taiwan; wchena.5253@mmh.org.tw (W.-C.C.); chchung@mmc.edu.tw (C.-H.C.); 3 Department of Nutritional Science, Fu Jen Catholic University, New Taipei City 252, Taiwan; 078670@mail.fju.edu.tw; 4 Department of Orthopedic Surgery, MacKay Memorial Hospital, Taipei 104, Taiwan; ckrisc@hotmail.com; 5 Department of Medical Research, MacKay Memorial Hospital, New Taipei City 252, Taiwan; d48906003@yahoo.com.tw; 6 Department of Gastroenterology, Hsinchu MacKay Memorial Hospital, Hsinchu City 300, Taiwan; pochuanaichen@yahoo.com.tw; 7 National Research Institute of Chinese Medicine, Ministry of Health and Welfare, Taipei 104, Taiwan; d91443006@ntu.edu.tw; 8 Pharmacological Institutes, College of Medicine, National Taiwan University, Taipei 104, Taiwan; jodiehsu779@hotmail.com; 9 Graduate Institute of Natural Products, College of Pharmacy, Kaohsiung Medical University, Kaohsiung 807, Taiwan

**Keywords:** hepatocellular carcinoma (HCC), metastasis, mosatilin, urokinase plasminogen activator (uPA)

## Abstract

Hepatocellular carcinoma (HCC) frequently shows early invasion into blood vessels as well as intrahepatic metastasis. Innovations of novel small-molecule agents to block HCC invasion and subsequent metastasis are urgently needed. Moscatilin is a bibenzyl derivative extracted from the stems of a traditional Chinese medicine, orchid *Dendrobium loddigesii*. Although moscatilin has been reported to suppress tumor angiogenesis and growth, the anti-metastatic property of moscatilin has not been elucidated. The present results revealed that moscatilin inhibited metastatic behavior of HCC cells without cytotoxic fashion in highly invasive human HCC cell lines. Furthermore, moscatilin significantly suppressed the activity of urokinase plasminogen activator (uPA), but not matrix metalloproteinase (MMP)-2 and MMP-9. Interestingly, moscatilin-suppressed uPA activity was through down-regulation the protein level of uPA, and did not impair the uPA receptor and uPA inhibitory molecule (PAI-1) expressions. Meanwhile, the mRNA expression of uPA was inhibited via moscatilin in a concentration-dependent manner. In addition, the expression of phosphorylated Akt, rather than ERK1/2, was inhibited by moscatilin treatment. The expression of phosphor-IκBα, and -p65, as well as κB-luciferase activity were also repressed after moscatilin treatment. Transfection of constitutively active Akt (Myr-Akt) obviously restored the moscatilin-inhibited the activation of NF-κB and uPA, and cancer invasion in HCC cells. Taken together, these results suggest that moscatilin impedes HCC invasion and uPA expression through the Akt/NF-κB signaling pathway. Moscatilin might serve as a potential anti-metastatic agent against the disease progression of human HCC.

## 1. Introduction

Hepatocellular carcinoma (HCC) is the most common malignancy of liver cancer and is the secondary cause of cancer-related death in the world, especially in Asian countries [1,2]. Generally, the risk factors of HCC are region, gender, and etiology. The male population, Asian people, and those infected by hepatitis virus B or C (HBV or HCV) are at a high-risk of HCC [3]. Early diagnosis and surgical resection are the better strategy for HCC therapy, which increase overall and disease-free survival rate [4]. However, the high potential of recurrences and distant metastasis enlarge the mortality rate of HCC. Therefore, developing a novel strategy for HCC treatment is necessary. 

Metastasis is a process that includes cell motility, local tumor cell invasion, intravasation and survival in blood circulation, extravasation, and growth at distant organs [5]. Degradation of the extracellular matrix (ECM) is an important and essential step of cancer cell metastasis. Numerous proteolytic enzymes such as matrix metalloproteinases (MMPs), and urokinase-type plasminogen activator (uPA)/uPA receptor (uPAR) system are involved in EMC degradation and the promotion of cell invasion [6,7]. The most important MMPs are gelatinase-A (MMP-2) and gelatinase-B (MMP-9) which degrades the type IV collagen and disconnects the cell from the basement membrane resulting in the increase of cell motility [6,8]. Overexpression or highly activation of MMP-2 and -9 to promote cancer cell metastasis has been observed in many cancer cells, such as breast, brain, prostate cancer and HCC [9,10,11,12]. In addition, serine proteinase uPA is also a critical molecule to involve in cancer cell metastasis. In normal physiological conditions, the PA system converts the plasminogen to plasmin and then destroys clots formation by the degradation of fibrins [13]. Two types of PA have been identified, tissue type of PA (tPA) and uPA. The function of tPA is to regulate clot lysis and synthesizes by epithelial cells [14]. Although uPA also involves in fibrinolysis, the predominant role of uPA is associated with tumor progression [15]. uPA is synthesized and secreted as a glycosylated zymogen, pro-uPA, and then activate by proteases cleavages, such as trypsin, plasmin, and cathepsin B and L [16,17]. After activation, uPA catalyzes plasminogen to plasmin which degrades ECM components, such as laminin, fibronectin and collagen and follows by prompting cell metastasis [18]. Furthermore, uPA also binds to uPAR and enhances its ability to plasmin formation and accelerate ECM degradation [19]. Interestingly, high uPA expression has been observed in prostate cancer patients compared with those with benign prostatic hyperplasia [20]. Overexpression of uPA and uPAR in many tumor samples are correlated with poorer pathological grade and shorter survival time [21,22,23,24]. Furthermore, overexpression of uPA mRNA in HCC has been indicated to increase invasiveness, metastasis and poor prognosis [25]. Therefore, targeting uPA might be a therapeutic strategy for the prevention of cancer invasion and metastasis and the improvement of cancer patient’s survival, especially in HCC.

Suppression of cancer cells’ metastatic abilities by traditional Chinese medicines is a strategy for preventing and/or treating cancer. Moscatilin (4,4′-dihydroxy-3,3′,5-trimethoxybibenzyl), a bibenzyl compound from orchid *Dendrobium loddigesii*, has been indicated to possess many biological activities, such as anti-inflammation, anti-oxidation, anti-angiogenesis, anti-proliferation and anti-metastasis [26,27,28,29,30,31]. Suppression of Akt-mediated epithelial-to-mesenchymal transition (EMT) via moscatilin in lung and triple-negative breast cancer cells has been demonstrated [30,31]. Furthermore, reactive oxygen species (ROS)-induced lung cancer cell invasion through the activation of focal adhesion kinase (FAK) and Akt is also repressed by moscatilin [27]. Although the anti-metastasis activities of moscatilin have been examined, the role of moscatilin in proteolytic enzyme-regulated cancer cells metastasis is still a mystery, especially in uPA-regulated HCC metastasis. In the present study, we examined the role of moscatilin in uPA-mediated HCC metastasis. Our results revealed that moscatilin suppresses uPA-mediated HCC metastasis via inhibiting the Akt-dependent NF-κB signaling pathway.

## 2. Results

### 2.1. Moscatilin Inhibited Metastatic Behavior of HCC Cells In Vitro and In Vivo

**Figure 1:**
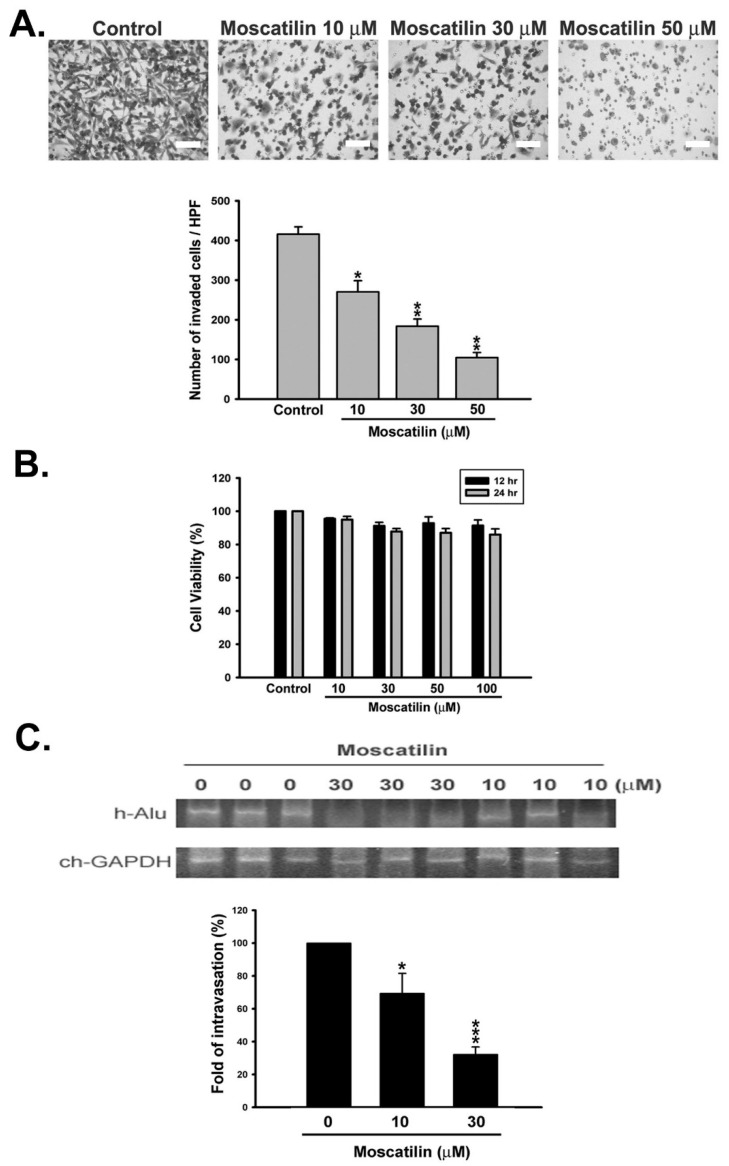
Effect of moscatilin on metastatic function in human hepatocellular carcinoma (HCC) cells. (**A**) SK-Hep-1 cells were seeded onto the upper chamber coated with Matrigel, then treated without or with moscatilin (10, 30, 50 μM) for 12 h in medium containing 10% FBS as a chemoattractant in the lower chamber. (**B**) SK-Hep-1 cells were treated with the indicated concentrations of moscatilin for 12 and 24 h in medium containing 10% FBS, and the cell viability was determined using MTT assay. (**C**) SK-Hep-1 cells were for treated with the indicated concentrations of moscatilin for 12 h and then subjected to CAM-intravasation assay coupled with the Alu PCR-based assay. The human HCC cells in the chick embryo lower CAM were determined using PCR of the human genome-specific Alu sequence with chick GAPDH specific primers as the control. Data are expressed as mean ± S.E.M. of four independent experiments. * *p* < 0.05, ** *p* < 0.01, and *** *p* < 0.001 compared the control group. Scale bar, 50 μm.

We first applied Transwell chamber assay and chick chorioallantoic membrane (CAM) intravasation model to explore the anti-invasive effect of moscatilin in vitro and in vivo, respectively. In Transwell chamber assay, human serum was used as a chemoattractant to induce in vitro invasion of SK-Hep-1 and HA22T cells. The results revealed that moscatilin significantly inhibited serum-induced cell invasion in a concentration-dependent manner (Figure 1A and Figure S1A). We also performed MTT assay to analyze the cytotoxicity of moscatilin in SK-Hep-1 cells. The cytotoxicity of moscatilin was still limited even in prolonged (24 h) and high concentration (100 μM) treatment (Figure 1B). These results demonstrated that moscatilin dramatically attenuates the invasion of HCC cells in the absence of cytotoxicity (Figure 1 and Figure S1). We further confirmed the anti-invasive activity of moscatilin on HCC cells by an in vivo CAM intravasation model. SK-Hep-1 cells were treated with the indicated concentration of moscatilin before being seeded on the upper CAM. After treatment, invading cells on the lower CAM were determined by human Alu gene (h-Alu) expression. As shown in Figure 1C, the expression of the h-Alu gene was significantly suppressed by moscatilin, indicating moscatilin inhibited the in vivo invasion activity of HCC cells. Collectively, our findings indicate that moscatilin exhibits promising anti-metastatic properties in human HCC cells.

### 2.2. Moscatilin Decreased the Proteolytic Activity of uPA

**Figure 2:**
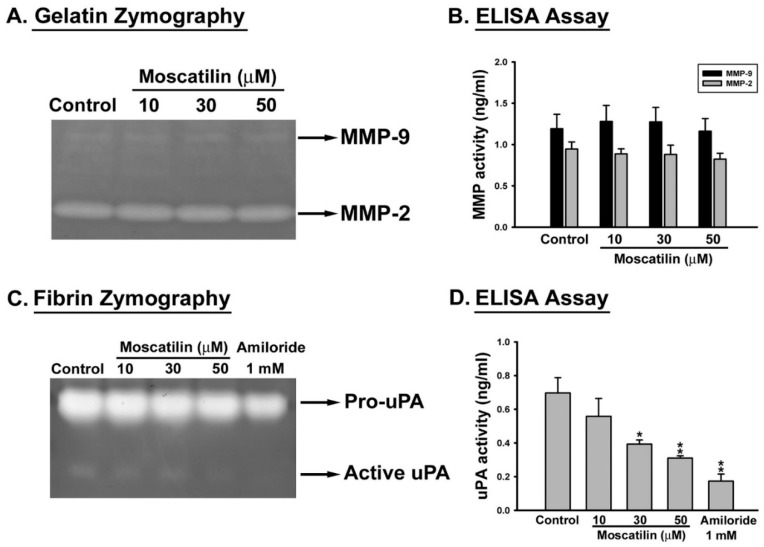
Effect of moscatilin on the enzyme activity of MMP and uPA in human HCC cells. SK-Hep-1 cells were treated without or with the indicated concentration of moscatilin or amiloride (1 mM) in medium containing 10% FBS. After 12 h of treatment, each conditioned medium was collected for analysis of proteolytic activities of MMP-2, MMP-9 and urokinase plasminogen activator (uPA) by zymography (**A**,**C**) and ELISA assay (**B**,**D**), respectively. Data are expressed as mean ± S.E.M. of four independent experiments. * *p* < 0.05, and ** *p* < 0.01 compared with the control group.

Matrix metalloproteases (MMPs)-2, -9, and urokinase plasminogen activator (uPA) are the key enzymes that participated in cell migration, invasion as well as metastasis [6,7]. To evaluate the role of these enzymes on moscatilin-inhibited cell invasion, SK-Hep-1 cells were treated with moscatilin (10–50 μM) or 1 mM amiloride (as the positive control for uPA) for 12 h and the enzyme activities of MMP-2, -9 and uPA was detected by zymography and quantified by ELISA. Suppression of MMP-2 and MMP-9 enzyme activities were not observed in moscatilin-treated cells (Figure 2A,B). Interestingly, the activity of uPA was significantly repressed by moscatilin in a concentration-dependent mode (Figure 2C,D). These results suggest that the inhibitory effect of moscatilin on HCC invasion might be through the down-regulation of uPA activity.

### 2.3. Moscatilin Suppressed uPA Expression on the Transcriptional Level

**Figure 3:**
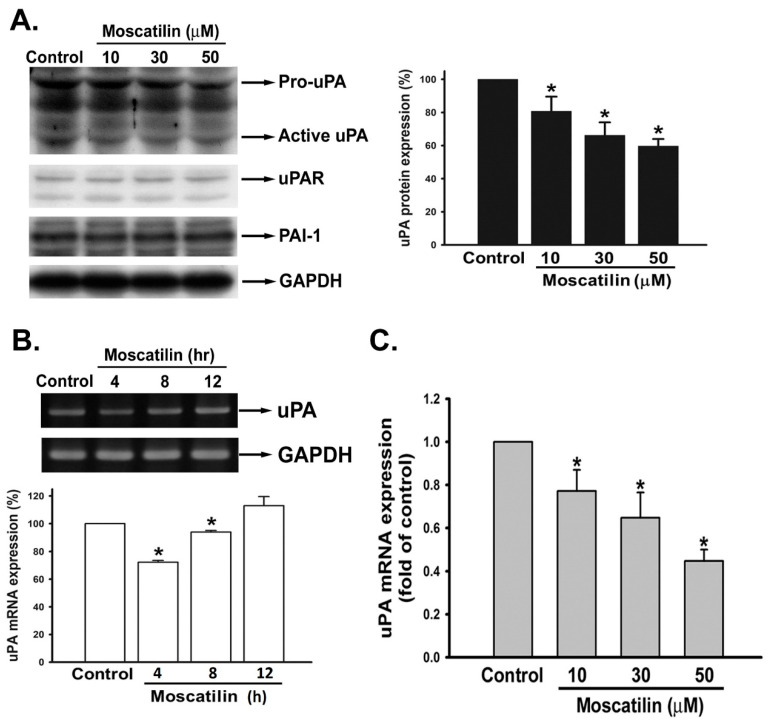
Effect of moscatilin on uPA mRNA and protein expression in human HCC cells. (**A**) SK-Hep-1 cells were treated with the indicated concentrations of moscatilin for 12 h in medium containing 10% FBS, and uPA, uPAR, and PAI-1 were determined by Western blot analysis. (**B**) SK-Hep-1 cells were treated with 30 μM of moscatilin for 4, 8, and 12 h. Then, the mRNA level of uPA was detected by RT-PCR. Imaging was performed using quantitative densitometry with Image-Pro Plus. (**C**) The quantitative real time PCR was performed after 4 h of incubation with moscatilin (10, 30, 50 μM) and normalized by GAPDH. Data are expressed as mean ± S.E.M. of four independent experiments. * *p* < 0.05 compared with the control group.

To further verify whether moscatilin represses uPA activity via transcription or translation alteration, we first analyzed the translation levels of uPA and its associated regulation molecules. SK-Hep-1 cells were treated with or without moscatilin (10–50 μM) for 12 h and the protein levels of uPA, uPAR, and PAI-1 (the uPA inhibitory molecule) were examined by Western blotting. Upon treatment with moscatilin, the active form of uPA was significantly down-regulated in a concentration-dependent mode. However, the protein levels of uPAR, as well as PAI-1, were not changed by moscatilin treatment (Figure 3A). We further analyzed the mRAN level of uPA. SK-Hep-1 cells were treated with 30 μM of moscatilin for 4, 8, and 12 h. Total RNA were extracted from treated SK-Hep-1 cells then reverse transcripted into cDNA for the semi-quantification of uPA on mRNA level. The expression of uPA mRNA in SK-Hep-1 cells was significantly reduced after treating moscatilin for 4 h (Figure 3B). Furthermore, the mRNA level of uPA was down-regulated in a concentration-dependent manner after moscatilin treatment (Figure 3C). The results implicate that moscatilin may inhibit HCC invasion by suppressing mRNA transcription of uPA.

### 2.4. Moscatilin Reduced Akt Rather Than ERK1/2 Phosphorylation

**Figure 4:**
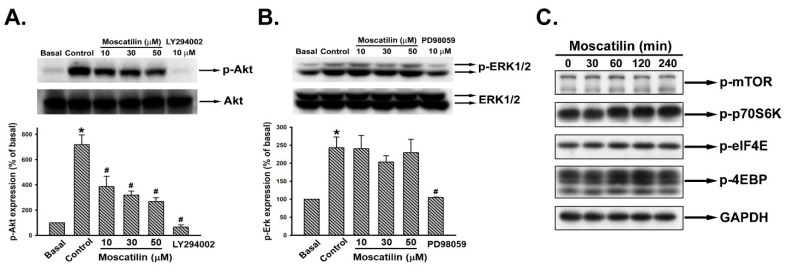
Effect of moscatilin on Akt, ERK, and translation pathways in human HCC cells. Serum-starved SK-Hep-1 cells were treated with vehicle (basal) or 10% FBS in the absence (control) or presence the indicated agents for 10 min. Cells were harvested and lysed for the detection of p-Akt (**A**) and p-ERK1/2 (**B**) by Western blot analysis. (**C**) SK-Hep-1 cells were treated with moscatilin (30 μM) for the indicated time intervals, and p-mTOR, p-p70S6K, p-eIF4E, and p-4EBP were determined by Western blot analysis. The quantitative densitometry of the relative level of protein was performed with Image-Pro Plus. Data are expressed as mean ± SEM of five independent experiments. * *p* < 0.05 compared with the basal group. # *p* <0.05 compared with the control group.

The upstream of uPA gene expression is involved with Akt and ERK1/2 signaling [32,33]. The activation status of Akt and ERK1/2 upon moscatilin treatment were assayed. Serum-starved SK-Hep-1 cells were treated with vehicle (basal), 10% FBS only (control), and 10% FBS containing moscatilin (10–50 μM), Akt inhibitor (LY294002), or ERK inhibitor (PD98059) for 10 min. Cell lysates were harvested and the phosphorylation of Akt (p-Akt) and ERK1/2 (p-ERK1/2) were analyzed. The expression of p-Akt was reduced upon moscatilin supplement (Figure 4A and Figure S2). In contrast to Akt, moscatilin did not affect ERK1/2 activation (Figure 4B). Further analysis of the expression of phosphorylated mTOR and the relative translation machinery molecules revealed that no significant repression was observed on the mTOR-mediated translational pathway in moscatilin-treated cells (Figure 4C).

### 2.5. Moscatilin Repressed the Activation of NF-κB

**Figure 5:**
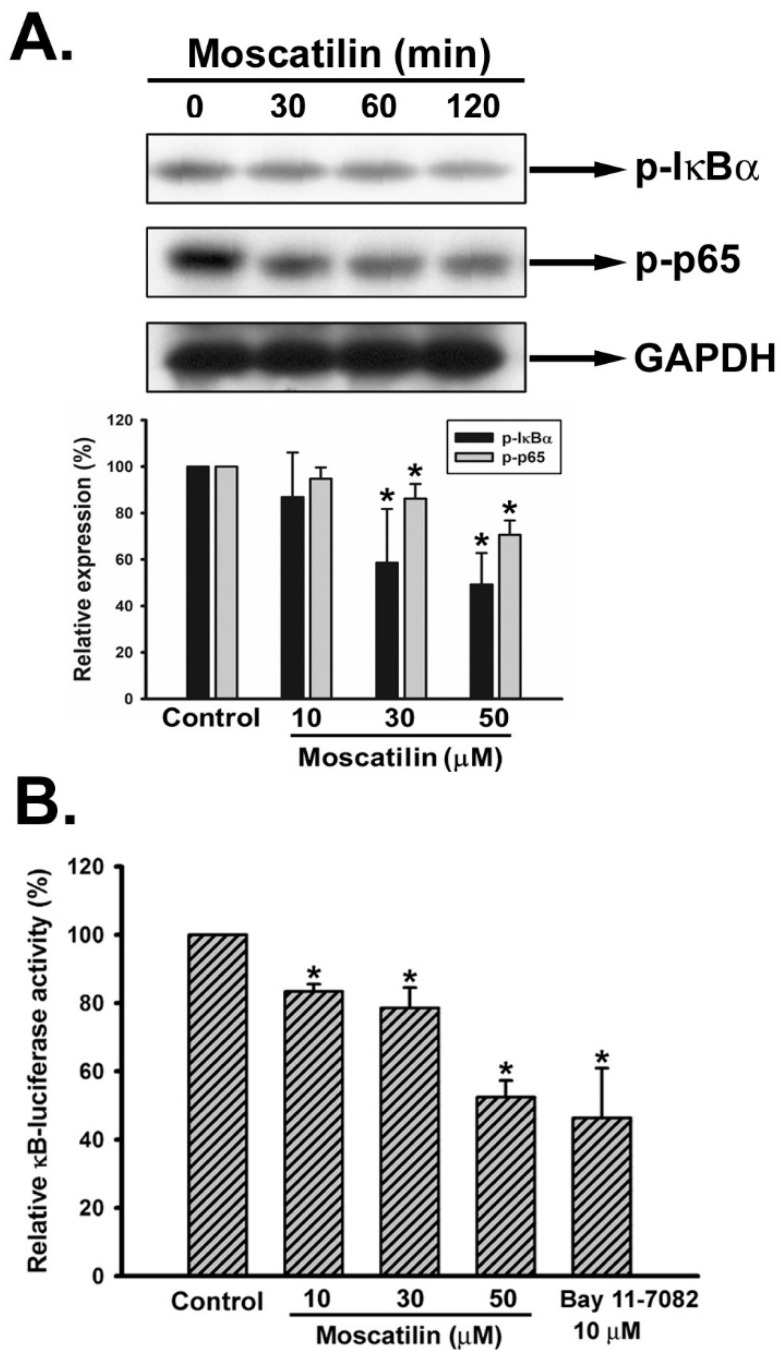
Effect of moscatilin on NF-κB pathway in human HCC cells. (**A**) SK-Hep-1 cells were treated with moscatilin (30 μM) for the indicated time intervals, and p-IκBα and p-p65 were determined by Western blot analysis. The quantitative densitometry of the relative level of protein was performed with Image-Pro Plus. (**B**) SK-Hep-1 cells cotransfected with pNF-κB-Luc and phRG-TK vector were treated with the indicated concentration of moscatilin or BAY 11-7082 for 12 h, and the promoter luciferase activities were detected using a luminometer. Data are expressed as means ± S.E.M. of four independent experiments. * *p* < 0.05 compared with the control group.

It has been documented that the up-regulation of uPA is regulated by nuclear factor-κB (NF-κB), a downstream molecule of Akt signaling [34]. Hence, we analyzed the activation of NF-κB signaling pathway upon moscatilin treatment. SK-Hep-1 cells were treated with 30 μM of moscatilin for different time intervals (0, 30, 60, 120 min) and the expression of phosphorylated IκBα (p-IκBα), phosphorylated p65 (p-p65) were evaluated. It was observed that both p-IκBα and p-p65 were reduced upon moscatilin treatment (Figure 5A). To further evaluate the role of NF-κB signaling pathway in moscatilin-suppressed uPA expression, pNF-κB-Luc reporter plasmid was administrated to confirm the inhibitory effect of moscatlin on NF-κB signaling. SK-Hep-1 cells were treated with moscatilin (10–50 μM) or the IκBα inhibitor (Bay 11-7082) for 12 h after transfection with pNF-κB-Luc plasmid. The data showed that the luciferase activity was significantly reduced upon moscatilin treatment (Figure 5B).

### 2.6. Moscatilin Impeded uPA Activation through Akt/NF-κB Signaling Pathway

**Figure 6:**
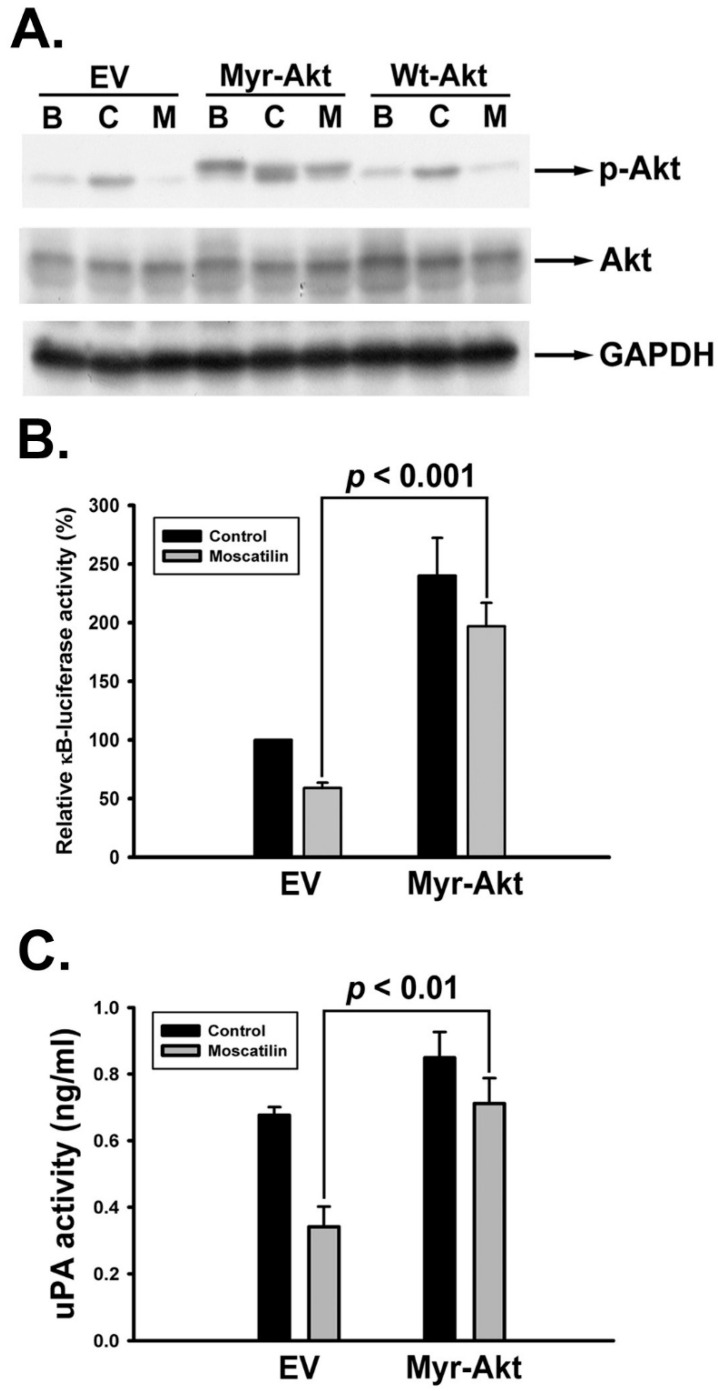
Involvement of Akt/NF-κB signaling pathway in moscatilin-suppressed uPA activity in human HCC cells. (**A**) SK-Hep-1 cells-transfected with empty vector (EV) or Myr-Akt or Wt-Akt were treated with vehicle (basal, B) or 10% FBS in the absence (control, C) or presence of moscatilin (30 μM, M) for 10 min. Cells were harvested and lysed for the detection of p-Akt by Western blot analysis. The quantitative densitometry of the relative level of protein was performed with Image-Pro Plus. SK-Hep-1 cells cotransfected with pNF-κB-Luc and Myr-Akt were treated with moscatilin (30 μM) for 12 h, and NF-κB luciferase activity (**B**) and uPA activity (**C**) were determined using reporter gene assay and ELISA assay, respectively. Data are expressed as mean ± S.E.M. of four independent experiments.

**Figure 7:**
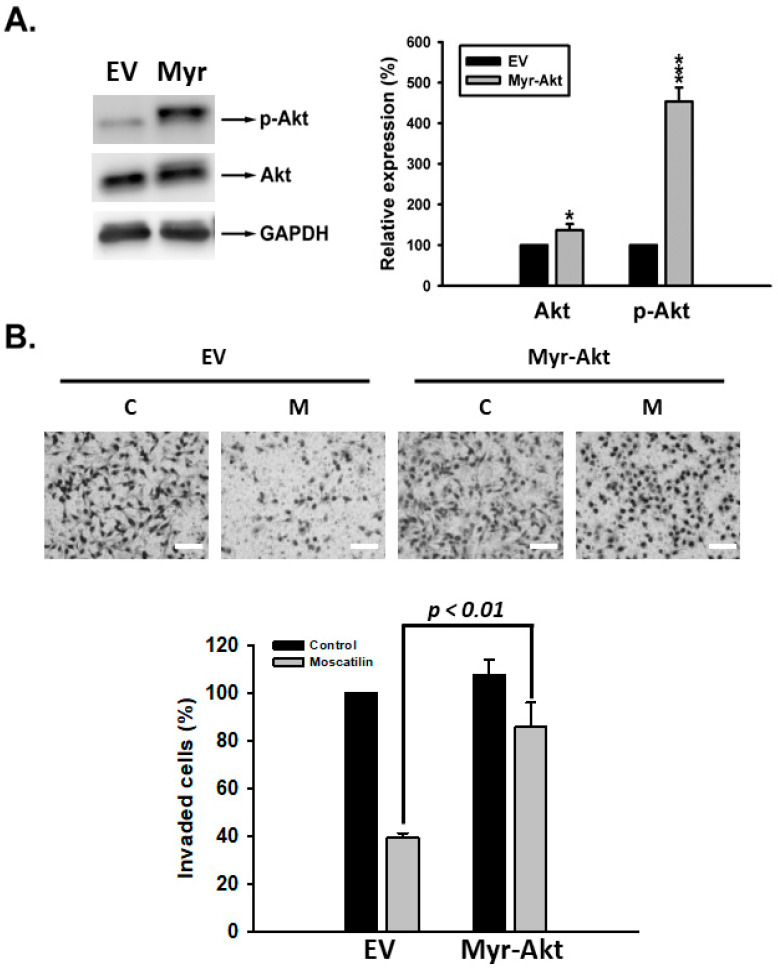
Overexpression of Akt prevents moscatilin-induced anti-invasive function. (**A**) SK-Hep-1 cells-transfected with empty vector (EV) or Myr-Akt (Myr) were harvested and lysed for the detection of p-Akt by Western blot analysis. (**B**) Transfected cells were seeded onto the upper chamber coated with Matrigel, then treated with (30 μM, M) or without (control, C) moscatilin for 12 h in medium containing 10% FBS as a chemoattractant in the lower chamber. Data are expressed as mean ± S.E.M. of four independent experiments. * *p* < 0.05, and *** *p* < 0.001 compared with the EV-transfected group. Scale bar, 50 μm.

Our results revealed that activation of Akt and NF-κB were inhibited upon moscatilin treatment (Figure 4A and Figure 5). Therefore we further verified whether Akt and NF-κB signals were involved in moscatilin-suppressed uPA activity. SK-Hep-1 cells were transiently transfected with empty vector (EV), constitutively activate myristoylated Akt (Myr-Akt) and wild-typed Akt (Wt-Akt) plasmid. After transfection, cells were then treated with vehicle, 10% FBS or moscatilin for 10 min and then the expression of phosphorylated Akt was detected. As shown in Figure 6A, overexpression of WT-Akt only have limited effect on the Akt-phosphorylation inhibiting ability of moscatilin. However, the transfection of Myr-Akt plasmid was able to retain the phosphorylation of Akt upon moscatilin treatment. Subsequently, moscatilin-inhibited NF-κB luciferase activity was rescued by Myr-Akt transfection (Figure 6B). Meanwhile, moscatilin-repressed uPA activity was also restored by Myr-Akt transfection (Figure 6C). Finally, Myr-Akt transfection significantly abrogated the anti-invasive activity of moscatilin (Figure 7). Taken together, we suggest that moscatilin may suppress HCC invasion by inhibiting uPA activation through Akt/NF-κB signaling pathway.

## 3. Discussion

**Figure 8:**
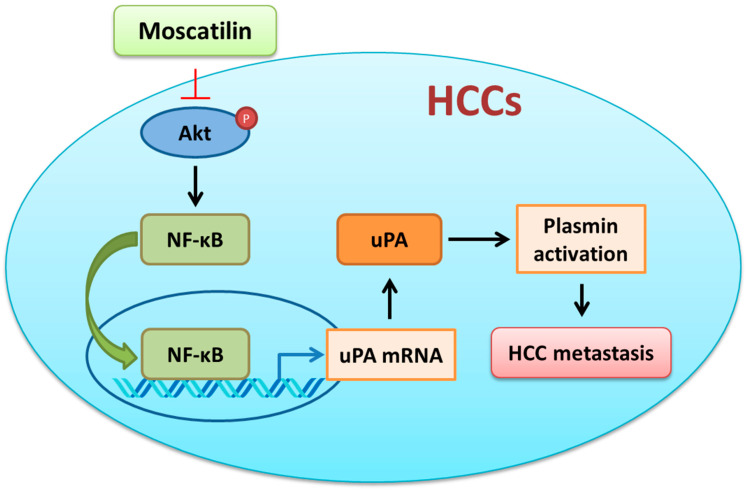
Illustrated model for our proposed molecular mechanism of how moscatilin inhibits HCC cell metastasis through inhibiting uPA expression via down-regulating Akt/NF-κB signaling pathway.

HCC is the most frequent liver cancer and the third leading cause of cancer-related death worldwide. Although systemic chemotherapeutic strategy has been applied for HCC, only 15-30% of late HCC patients noticeably improve the average survival rate [35]. Furthermore, early invasion to distant organs and high potential of recurrence rate also makes HCC hard to cure. Thus, searching for novel small-molecule agents to suppress cancer cell metastasis might be a promising strategy for HCC treatment. In the present study, we examined the effects of moscatilin on HCC metastasis. The results revealed that the invasion activity of SK-Hep-1 cells was suppressed by moscatilin at in vitro and in vivo levels. Repression of uPA, rather than MMP-2 and -9, activity was observed in moscatilin-treated SK-Hep-1 cells. Furthermore, our results demonstrated that moscatilin suppressed the expression of uPA at translation level via inhibiting the Akt-mediated NF-κB activation in SK-Hep-1 cells (Figure 8). This study provides new evidences supporting the development of moscatilin against HCC cells.

The extracellular proteolytic enzyme system of PA involves in many physiological and pathophysiological processes [16,17]. Conversion of plasminogen to plasmin by activated uPA will lead to the initiation of metastatic cascade [36]. Furthermore, uPA and pro-uPA also bind to uPAR to trigger plasmin activation [37]. Neutralization of uPA via plasminogen activator inhibitor-1 and -2 (PAI-1 and -2) inhibits uPA-induced cell migration and invasion [38]. High expression of uPA, uPAR has been observed in HCC tumor tissues which is associated to increase tumor growth, metastasis, and poor prognosis of HCC [25]. Therefore, targeting the uPA system might be a good strategy for cancer therapy, especially for HCC. As shown in Figure 3, moscatilin down-regulated the expression of uPA at the transcription level but did not affect the expression of uPAR and PAI-1, implicating that down-regulation of uPA via moscatilin might be through transcriptional mechanism regulation. In normal conditions the expression of uPA is low. However, when the cells encounter stimulators such as growth factors, cytokines, steroid hormones, and genotoxic agents, the expression of uPA will dramatically increase [39]. Transcription factors, such as Sp-1, Ap-1, and NF-κB, have been indicated to be responsible for the activation of uPA expression upon stimulators induction [40]. Increased Sp-1 and Ap-1 binding activity via ERK1/2 is the predominant mechanism in the up-regulation of uPA [41,42]. In addition, NF-κB-mediated uPA up-regulation is also observed in PMA (phorbol 12-myristate 13-acetate)-stimulated HeLa and HepG2 cells [43]. Overexpression of uPA is regulated by constitutive activation of NF-κB p65 subunit in pancreatic adenocarcinomas [35]. Moreover, activation of NF-κB via Akt signaling has been indicated to participate in cancer cells invasion [44]. Recent studies have also shown that suppression of cancer cell migration and invasion via uPA inhibition is mediated through down-regulation of ERK1/2- and Akt-dependent signaling pathways [33,34,45]. In this study, we demonstrated that moscatilin only down-regulated the expression of NF-κB by inhibiting the phosphorylation of Akt. Furthermore, exogenous expression of a constitutively activated form of Akt can rescue the activity of NF-κB and uPA in the presence of moscatilin. Although down-regulation of ERK1/2-regulated cells metastasis was observed in moscatilin-treated lung cancer cell [27], no significant difference of ERK1/2 activation in moscatilin-treated HCC was detected. Furthermore, Akt-mediated translational mechanisms were also investigated to verify the role of moscatilin in uPA down-regulation. However, the expression of translational mechanism markers, such as p-mTOR, p-p70S6K, p-elF4E, and p-4EBP, were not affected by moscatilin. The results indicated that moscatilin-inhibited uPA was through Akt-mediated transcriptional regulation, rather than Akt-mediated translational mechanisms. 

Moscatilin is an active compound isolated from orchid *Dendrobium loddigesii* and possesses many biological activities. Recent studies have been indicated that moscatilin suppresses metastasis of triple-negative breast cancer cells and lung cancer cells without any observed in vivo toxicity highlighting the potential of moscatilin as a cancer treatment [28,30]. Furthermore, down-regulation of Akt-mediated EMT was also observed in moscatilin-treated lung cancer and breast cancer cells [30,31]. In addition, inhibition of NF-κB signaling pathway also leads to down-regulation of Twist-dependent pathway and the expression of COX-2 and iNOS [26,30]. Whether moscatilin inhibit the metastasis of HCC cell through these pathways requires further investigation. In conclusion, this is the first study that demonstrates moscatilin suppresses HCC metastasis through Akt/NF-κB-mediated uPA down-regulation, highlighting the potential of moscatilin as a future HCC treatment. 

## 4. Materials and Methods

### 4.1. Chemicals and Reagents

Moscatilin (Figure S3) was extracted and purified by one of our colleagues (Prof. Chien-Chih Chen) and the purity is more than 98%. Dulbecco’s modified Eagle’s medium (DMEM), fetal bovine serum (FBS), penicillin and streptomycin were acquired from Gibco (Grand Island, NY, USA). Anti-p-ERK1/2, -ERK1/2, -p-p70S6K, and anti-p-IκBα, were purchased from Cell Signaling Technologies (Boston, MA, USA). Anti-p-Akt, -Akt, -p-mTOR, -p-NF-κB/p65, and anti-GAPDH were obtained from Epitomics (Burlingame, CA, USA). Anti-mouse and anti-rabbit IgG-conjugated horseradish peroxidase and Anti-uPA, -uPAR, and anti-PAI-1 were obtained from Santa Cruz Biotechnology (Santa Cruz, CA, USA).

### 4.2. Cell Culture and Cell Viability Analysis

The human HCC cell line SK-Hep-1 (American Type Culture Collection, Manassas, VA, USA) and HA22T (Culture Collection and Research Center, Hsinchu, Taiwan) were maintained in DMEM containing 10% FBS, penicillin (100 units/mL), streptomycin (100 μg/mL), and L-glutamine (2 mM) at 37 °C in humidified air containing 5% CO_2_. For cell viability assay, SK-Hep-1 cells (10^4^ cells/well) were seeded in 96 well plates. After seeding for 24 h, cells were treated with a serial dosage of moscatilin for 24 h. Then, cell survival was determined by MTT assay.

### 4.3. In Vitro Invasion Analysis

In vitro cell invasion assay was performed as described previously [46]. Briefly, transwell inserts with 8 μm pore-size (Corning, Corning, NY, USA) were pre-coated with 30 μL of Matrigel (BD Biosciences, Bedford, MA, USA) for 30 min. Then HCC cells (5 × 10^4^ cells/100 μL) were seeded with serum-free media in the upper chamber, and 500 μL of 10% FBS-containing media in the absence or presence of moscatilin were treated in the lower chamber. After 12 h of treatment, the upper side of cells were removed by cotton-tipped swabs, and the lower side of cells were fixed and stained with 0.05% crystal violet for 15 min. The invaded cells were photographed and quantified by counting the number of stained cells under a microscope. 

### 4.4. In Vivo Invasion Analysis 

The in vivo metastasis was performed the chorioallantoic membrane (CAM) intravasation model and as described previously [46]. Briefly, SK-Hep-1 cells were treated with moscatilin for 12 h, surviving cells (10^6^ cells) were harvested and resuspended in serum-free DMEM and inoculated onto the CAM (upper CAM) of a 9-day-old chick embryo. The lower half of the CAM (lower CAM) was removed and stored frozen at −80 °C after 48 h of incubation. Genomic DNA of the frozen tissue was prepared from lower CAM. To determine human cells in the chick tissues, the primers specific for human Alu sequence (5′-ACGCCTGTAATCCCAGCACTT-3′/5′-TCGCCCAGGCTGGAGTGCA-3′) were used to amplify the human Alu repeats. A quantitative measure of amplifiable chick DNA was examined by amplification of chick GAPDH genomic DNA sequence with chGAPDH primers (5′-GAGGAAAGGTCGCCTGGTGGATCG-3′/5′-GGTGAGGACAAGCAGTGAGGAACG-3′). The cycling conditions were 10 min of polymerase activation at 95 °C followed by 30 cycles at 95 °C for 30 s, 58 °C for 45 s, and 72 °C for 45 s and a final incubation at 72 °C for 10 min. Finally, PCR products were conducted on the agarose gel and visualized using ethidium bromide.

### 4.5. MMP-2, 9 and uPA Activity Assay 

The activities of MMP-2, 9 and uPA in conditional medium were measured by zymography protease assays. After moscatilin treatment, the media were collected and stored at −80 °C. Briefly, collected media of an appropriate volume (adjusted by vital cell number) were prepared with SDS sample buffer without boiling or reduction and subjected to 8% SDS-PAGE containing 0.1% gelatin or 2% casein (*w*/*v*) and 20 µg/mL plasminogen electrophoresis for MMP-2 and -9, and u-PA activity, respectively. After electrophoresis, gels were washed with 2.5% Triton X-100 and incubated with reaction buffer (40 mM Tris–HCl, pH 8.0; 10 mM CaCl_2_ and 0.01% NaN_3_) for 12 h at 37 °C. Gel was stained with coomassie brilliant blue R-250. Further, to quantify the concentration of MMP-2, -9 and uPA in media, ELISA kits (R&D Systems, Minneapolis, MN, USA) were performed following the manufacturer’s protocol.

### 4.6. Western Blot Analysis

The moscatilin-treated cellular lysates were prepared according our previous instruction [46]. Briefly, proteins were separated by 8–12% SDS-PAGE and immunoblotted with specific primary and secondary antibodies. The signals were determined using the chemiluminescent assay kit (Amersham, Buckinghamshire, UK).

### 4.7. Reverse Transcription Polymerase Chain Reaction (RT-PCR) and TaqMan Quantitative Real-Time PCR Analysis 

Total RNA was extracted from cells by using a TRIzol kit (MDBio Inc., Taipei, Taiwan). cDNAs were prepared using random primer and moloney murine leukemia virus reverse transcriptase (M-MLV RT) following the manufacturer’s protocol. The primer sequences used for amplification were listed as follows: uPA, 5′-AGAATTCTACCGACTATCTC-3′/5′-ATTCTCTTCCTTGGTGTGAC-3′; GAPDH, 5′-TGATGACATCAAGAAGGTGGTGAAG-3′/5′-TCCTTGGAGGCCATGTGGGCCAT-3′. The amplification conditions were as follows: initial denaturation at 94 °C for 5 min, 30 cycles of amplification for uPA and GAPDH (94 °C for 60 s, 60 °C for 60 s, and 72 °C for 60 s) and a final extension step at 72 °C for 10 min. The PCR products were separated on the agarose gel and visualized using ethidium bromide. 

The quantitative real-time PCR analysis was conducted with a TaqMan one-step PCR Master Mix (Applied Biosystems, Foster City, CA, USA). 1 μg of total cDNA was added per 25 μL reactions with uPA or GAPDH primers and TaqMan probes. All quantitative real-time PCR assays were performed in a triplicate manner on a StepOnePlus sequence detection system. The threshold was set within the linear phase of uPA gene amplification and above the background control group to calculate the cycle number where the transcript was detected. 

### 4.8. Reporter Gene Assay 

Nuclear factor-κB (NF-κB) promoter plasmid transfection was performed using Lipofectmine 2000 (Invitrogen, Carlsbad, CA, USA) according to the manufacturer’s protocol. SK-Hep-1 cells were cotransfected with NF-κB promoter plasmid (pNF-κB-Luc) and Renilla luciferase reporter vector (phRGTK). After 24 h of transfection, cells were treated with moscatilin or BAY 11-7082 for 12 h. Cell extracts were then harvested, and the activities of firely and Renilla luciferases were detected by DLR assay system (Promega, Madison, WI, USA) using FlexStation 3 (Molecular Devices, San Jose, CA, USA). 

### 4.9. Plasmids and Transient Transfection Analysis

The pUSEamp-Akt1 cDNA (wild-type Akt, wt-Akt) and pUSEamp-myr-Akt1 cDNA (constitutively active Akt, myr-Akt) were gifts from Prof. Chien-Huang Lin (Taipei Medical University, Taipei, Taiwan). SK-Hep-1 cells (5 × 10^4^) were seeded into 24-well plates in standard growth medium. After an overnight culture, the cells were transfected with empty vector, Myr-Akt and Wt-Akt, and Lipofectamine 2000 (Invitrogen, Carlsbad, CA, USA) according to the manufacturer’s instruction. 8 h post-transfection, the medium was replaced with 10% FBS DMEM and further incubated overnight. Transfected cells were starved for 16 h before treated with moscatilin for the indicated time. After moscatilin treatment, Western blot, NF-κB promoter assay, uPA ELISA assay, and invasion analysis were conducted in transfected cells.

### 4.10. Statistical Analysis 

Data are presented as the mean ± standard error of mean (SEM). Statistical analyses of data were done with Student’s t test and one-way ANOVA with post host Bonferroni test. The significant differences were indicated when the *p* value is < 0.05.

## Data Availability

Not Applicable.

## Supplementary Materials

The following are available online at https://www.mdpi.com/1422-0067/22/6/2930/s1.
