# Supplementary material for: Moscatilin Inhibits Metastatic Behavior of Human Hepatocellular Carcinoma Cells: A Crucial Role of uPA Suppression via Akt/NF-κB-Dependent Pathway
Source: Int J Mol Sci. 2021 Mar 13;22(6):2930. doi: 10.3390/ijms22062930 (PMC8002083; doi:10.3390/ijms22062930)
Supplement: Supplementary file 1 [file ijms-22-02930-s001.pdf]

Supplementary Data

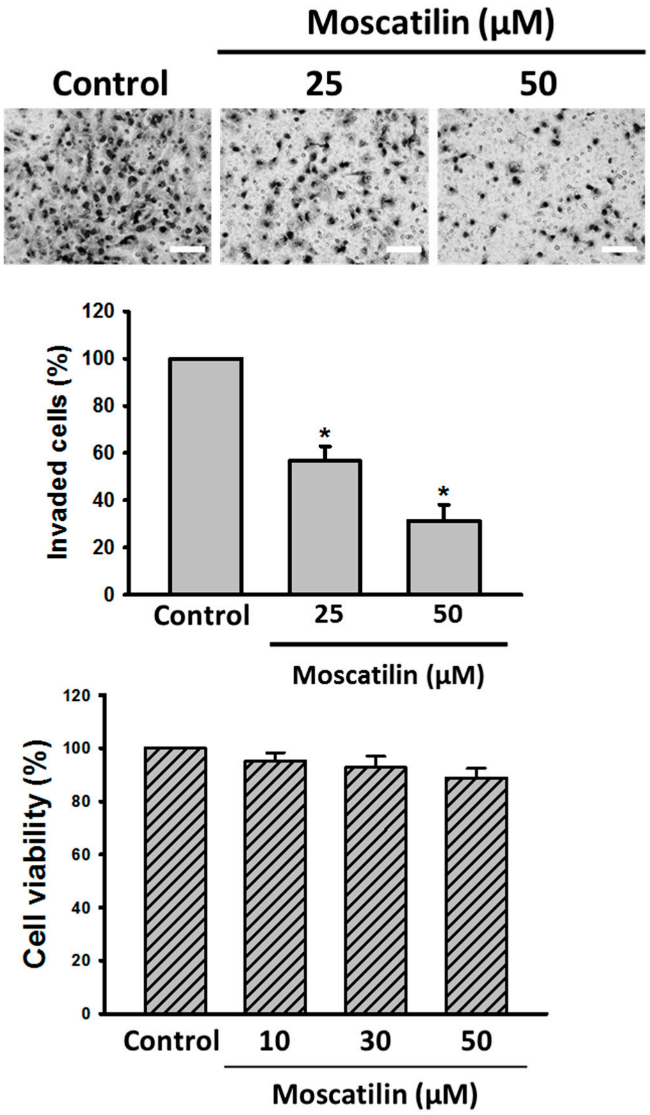

Supplementary Figure S1. Effect of moscatilin on cell invasion in HA22T cells. A, Cells were seeded onto the upper chamber coated with Matrigel, then treated without or with moscatilin (25, 50 μM) in medium containing 10% FBS as a chemoattractant in the lower chamber. B, HA22T cells were treated with the indicated concentrations of moscatilin in medium containing 10% FBS, and the cell viability was determined using MTT assay. Data are expressed as mean ± S.E.M. of three independent experiments . * $p < 0.05$ compared the control group. Scale bar, 50 μm.

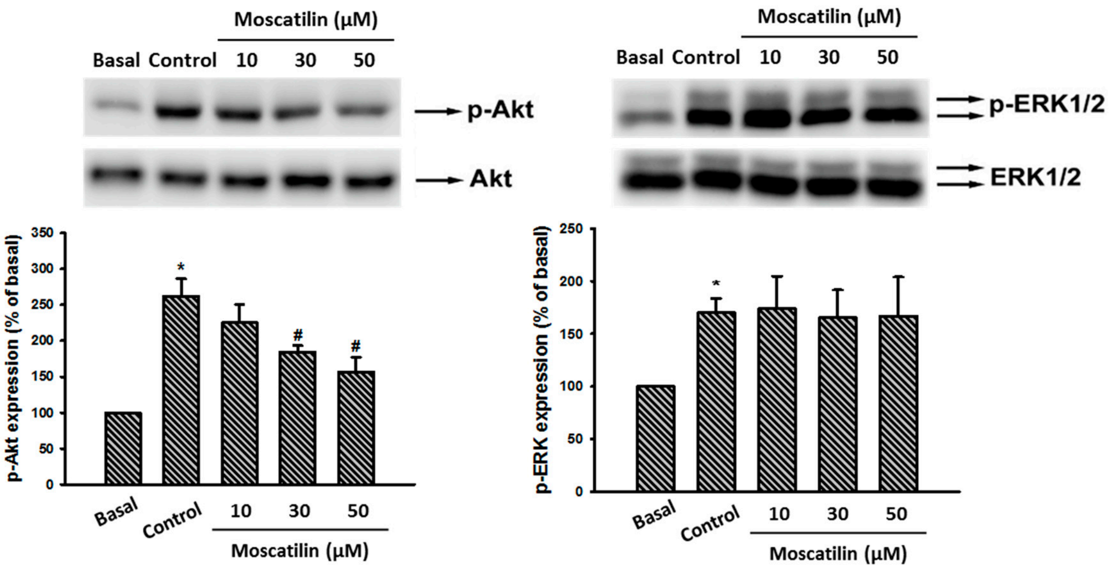

Supplementary Figure S2. Effect of moscatilin on the phosphorylation of Akt and ERK in HA22T cells. Serum-starved cells were treated with vehicle (basal) or 10% FBS in the absence (control) or presence the indicated agents for 10 min. Cells were harvested and lysed for the detection of p-Akt and p-ERK1/2 by Western blot analysis. The quantitative densitometry of the relative level of protein was performed with Image-Pro Plus. Data are expressed as mean ± SEM of three independent experiments. * $p < 0.05$ compared with the basal group. # $p < 0.05$ compared with the control group.

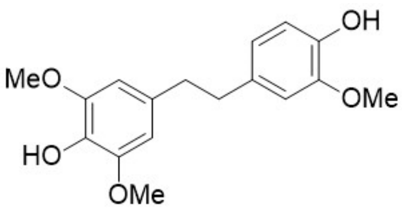

Supplementary Figure S3. Structure of moscatilin.
